# Supplementary material for: Concurrent maintenance of both veridical and transformed working memory representations within unique coding schemes
Source: Imaging Neurosci (Camb). 2024 May 20;2:imag-2-00173. doi: 10.1162/imag_a_00173 (PMC12247577; doi:10.1162/imag_a_00173)
Supplement: Supplementary Material [file imag_a_00173-supp.pdf]

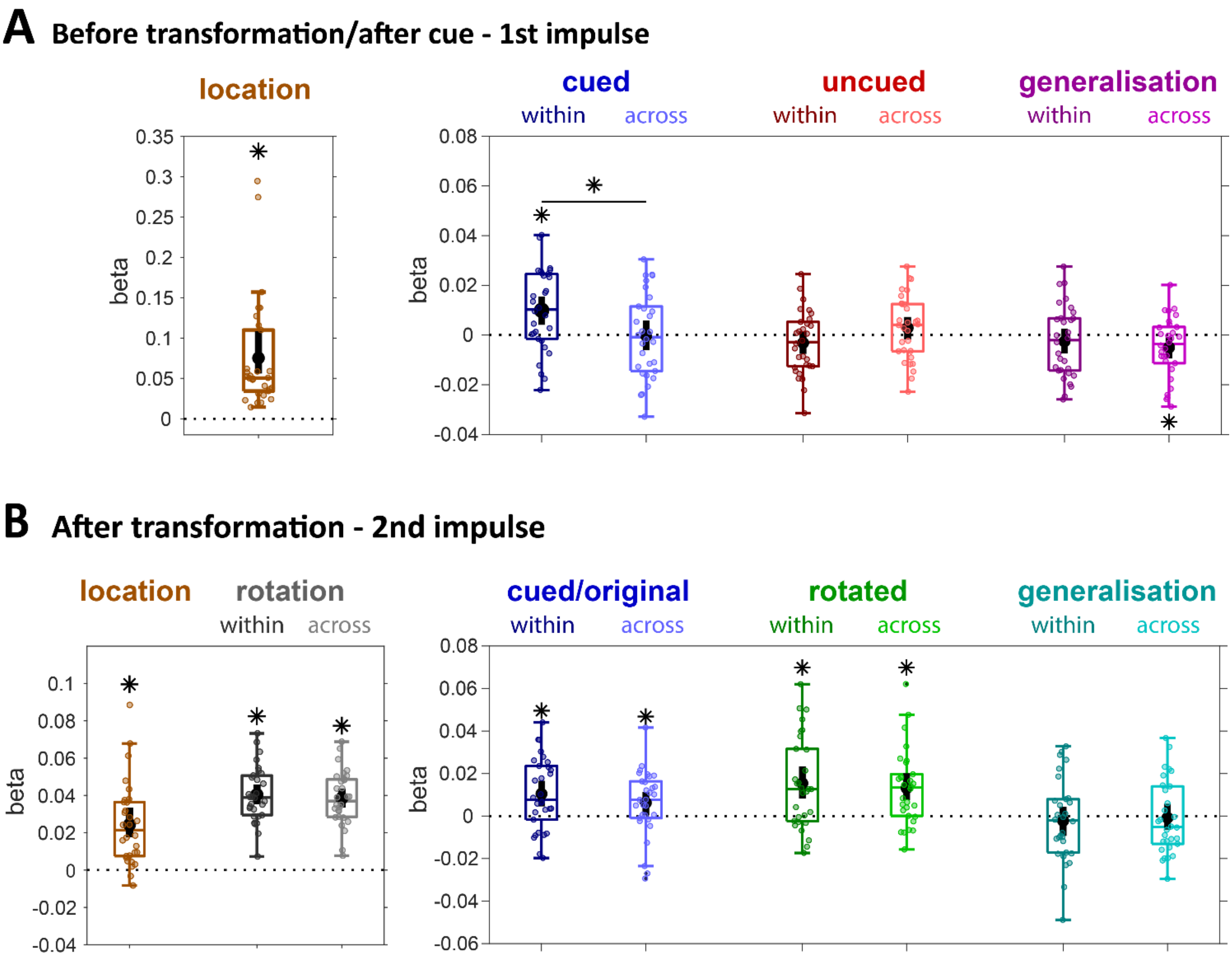

Supplemental Figure 1. LDC results of the spatiotemporal signal using a pre-trial baseline (-200 to 0 ms, relative to memory onset). **A.** Beta values of model fits for Impulse 1. Cued location model: $p < 0.01$, $BF_{10} > 1000$. Cued items models: within location, $p < 0.01$, $BF_{10} = 39.958$; across location, $p = 0.974$, $BF_{10} = 0.196$, difference; $p = 0.018$, $BF_{10} = 4.395$. Uncued item models: within, $p = 0.172$, $BF_{10} = 0.453$; across, $p = 0.246$, $BF_{10} = 0.423$; difference, $p = 0.084$, $BF_{10} = 1.365$. Generalisation models (cued/uncued): within, $p = 0.338$, $BF_{10} = 0.304$; across, $p = 0.032$, $BF_{10} = 1.946$; difference, $p = 0.406$, $BF_{10} = 0.259$. **B.** Beta values of model fits for Impulse 2. Cued location model: $p < 0.01$, $BF_{10} > 1000$. Rotation condition models: within location, $p < 0.01$, $BF_{10} > 1000$; across location, $p < 0.01$, $BF_{10} > 1000$, difference; $p = 0.826$, $BF_{10} = 0.354$. Cued items models: within location, $p < 0.01$, $BF_{10} = 37.709$; across location, $p = 0.042$, $BF_{10} = 1.655$, difference; $p = 0.456$, $BF_{10} = 0.258$. Rotated item models: within, $p < 0.01$, $BF_{10} = 85.023$; across, $p < 0.01$, $BF_{10} = 201.893$; difference, $p = 0.878$, $BF_{10} = 0.213$. Generalisation models (cued/rotated): within, $p = 0.548$, $BF_{10} = 0.255$; across, $p = 0.824$, $BF_{10} = 0.2$; difference, $p = 0.744$, $BF_{10} = 0.199$.

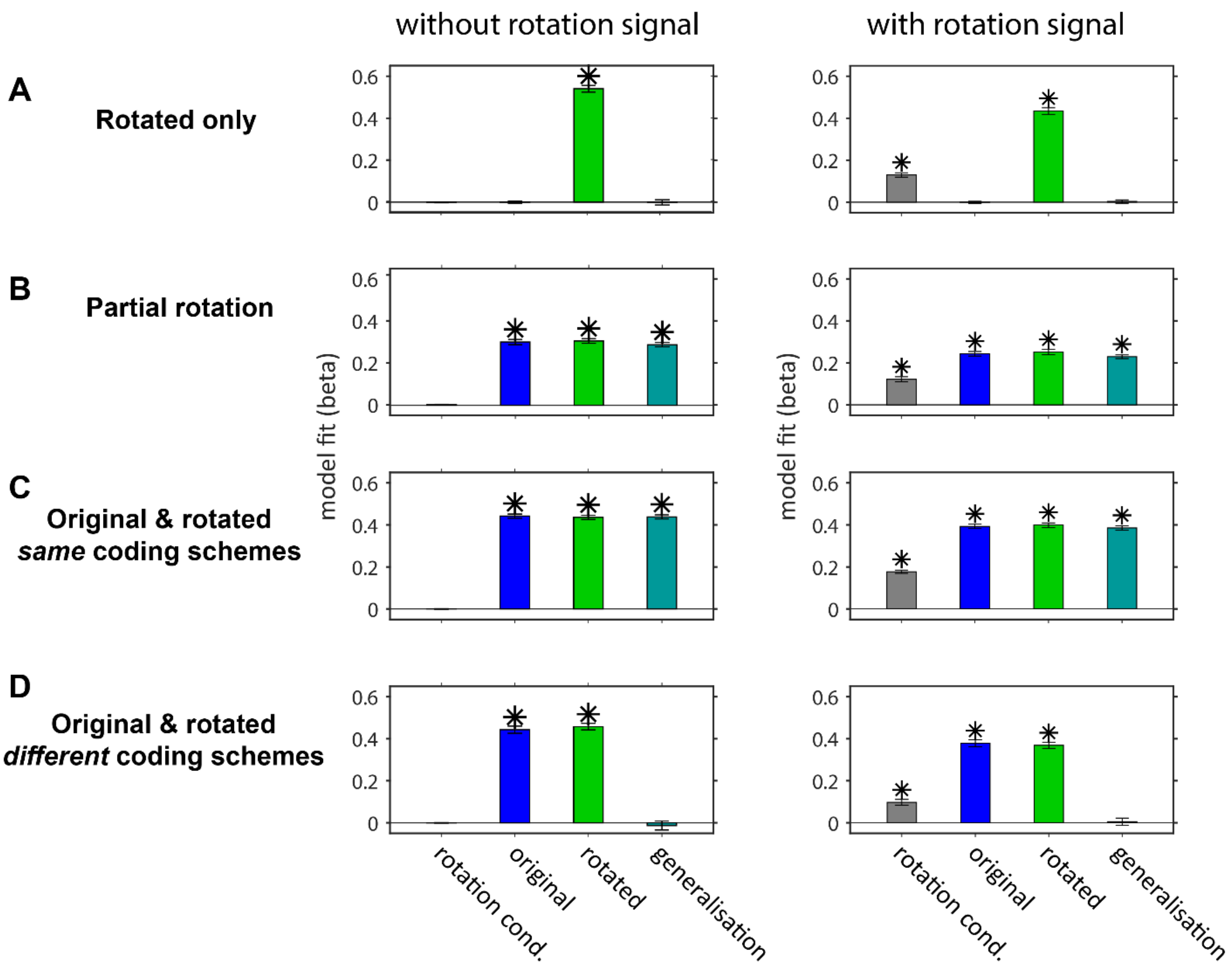

Supplemental Figure 2. Simulation results when an explicit rotation signal was not added (left column, as in Fig. 4), and when it was added (right column).

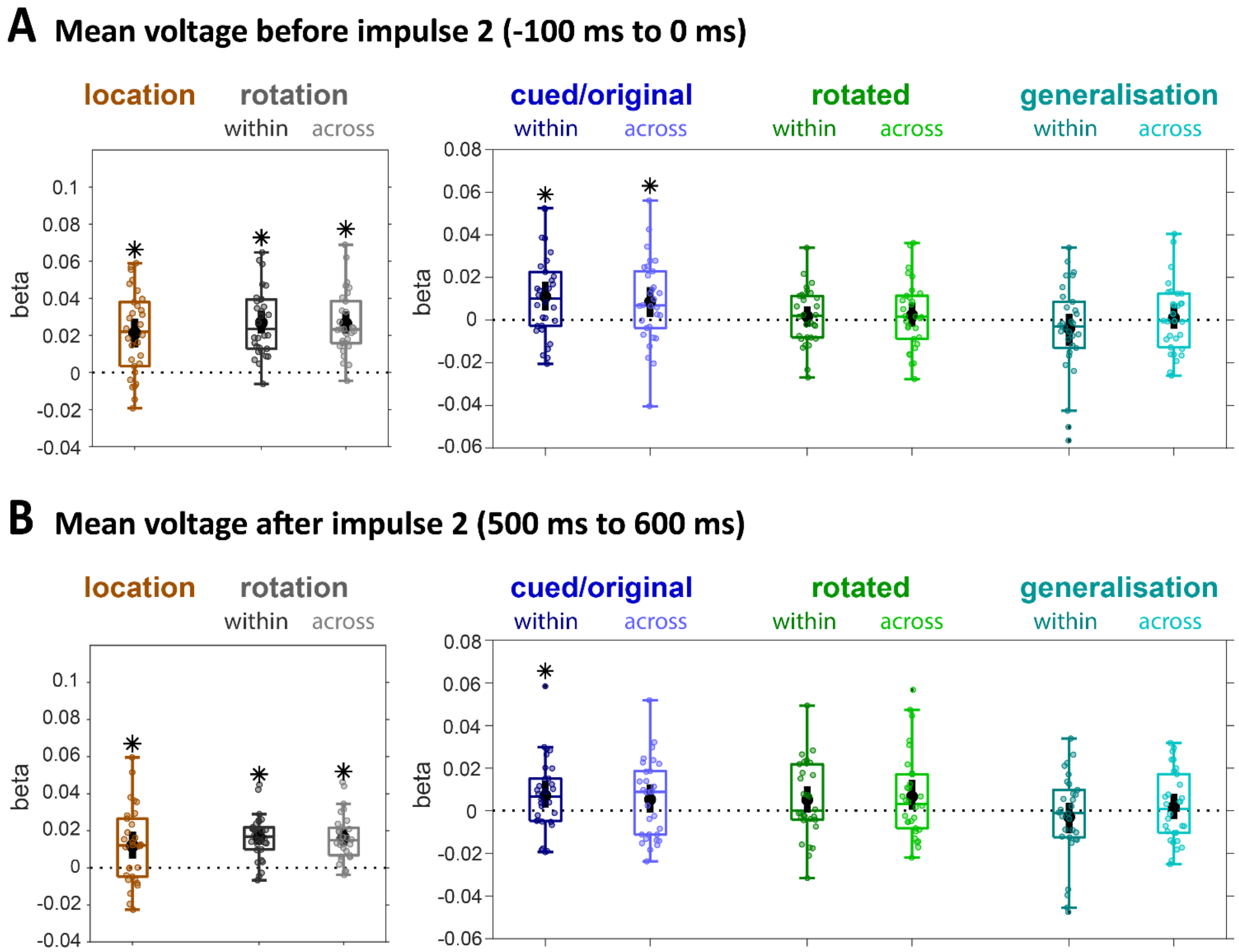

Supplemental Figure 3. LDC results at Impulse 2 of the mean voltage immediately before (-100 ms to 0 ms relative to Impulse 2), and after Impulse 2 presentation (500 ms to 600 ms relative to Impulse 2 and -100 ms to 0 relative to Probe onset), using a pre-trial baseline (-200ms to 0, relative to memory array onset). **A.** Beta values of model fits before Impulse 2. Cued location model: $p < 0.01$, $BF_{10} > 1000$. Rotation condition models: within location, $p < 0.01$, $BF_{10} > 1000$; across location, $p < 0.01$, $BF_{10} > 1000$, difference; $p = 0.876$, $BF_{10} = 0.204$. Cued items models: within location, $p < 0.01$, $BF_{10} = 17.248$; across location, $p = 0.03$, $BF_{10} = 4.179$, difference; $p = 0.636$, $BF_{10} = 0.201$. Rotated item models: within, $p = 0.448$, $BF_{10} = 0.272$; across, $p = 0.448$, $BF_{10} = 0.270$; difference, $p = 0.972$, $BF_{10} = 0.199$. Generalisation models (cued/rotated): within, $p = 0.312$, $BF_{10} = 0.314$; across, $p = 0.774$, $BF_{10} = 0.196$; difference, $p = 0.346$, $BF_{10} = 0.598$. **B.** Beta values of model fits after Impulse 2. Cued location model: $p < 0.01$, $BF_{10} = 24.482$. Rotation condition models: within location, $p < 0.01$, $BF_{10} > 1000$; across location, $p < 0.01$, $BF_{10} > 1000$, difference; $p = 0.692$, $BF_{10} = 0.204$. Cued items models: within location, $p = 0.044$, $BF_{10} = 3.259$; across location, $p = 0.126$, $BF_{10} = 0.763$, difference; $p = 0.616$, $BF_{10} = 0.208$. Rotated item models: within, $p = 0.098$, $BF_{10} = 0.565$; across, $p = 0.060$, $BF_{10} = 0.583$; difference, $p = 0.884$,

BF_{10} = 0.214. Generalisation models (cued/rotated): within, p = 0.448, BF_{10} = 0.248; across, p = 0.582, BF_{10} = 0.583; difference, p = 0.350, BF_{10} = 0.459.

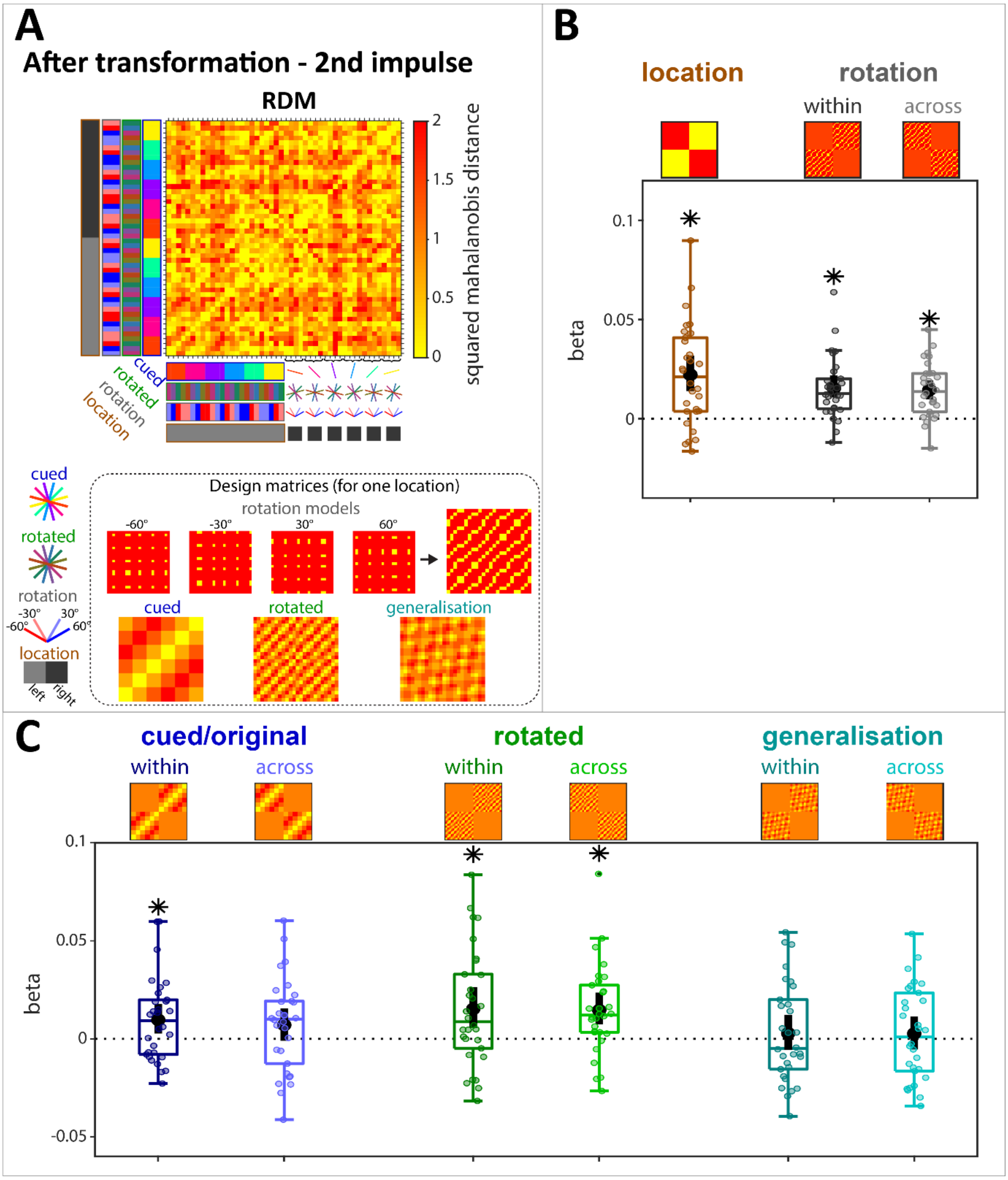

Supplemental Figure 4. LDC of the spatiotemporal signal at Impulse 2 when excluding all non-rotation trials. **A.** Average RDM and design matrices of models. **B.** Beta values of the cued location model ($p < 0.01$, $BF_{10} = 582.24$) and the rotation condition models (within and across location: $p < 0.01$, $BF_{10} > 1000$; difference: $p = 0.784$, $BF_{10} = 0.218$). **C.** Beta values of cued item models (within: $p = 0.026$, $BF_{10} = 4.502$; across: $p = 0.14$, $BF_{10} = 0.571$; difference: $p = 0.51$, $BF_{10} = 0.208$), rotated item models (within: $p = 0.014$, $BF_{10} = 7.339$; across: $p < 0.01$, $BF_{10} = 132.813$; difference: $p = 0.652$, $BF_{10} = 0.196$), and generalization models (within: $p = 0.538$, $BF_{10} = 0.239$; across: $p = 0.538$, $BF_{10} = 0.265$; difference: $p = 0.986$, $BF_{10} = 0.196$).

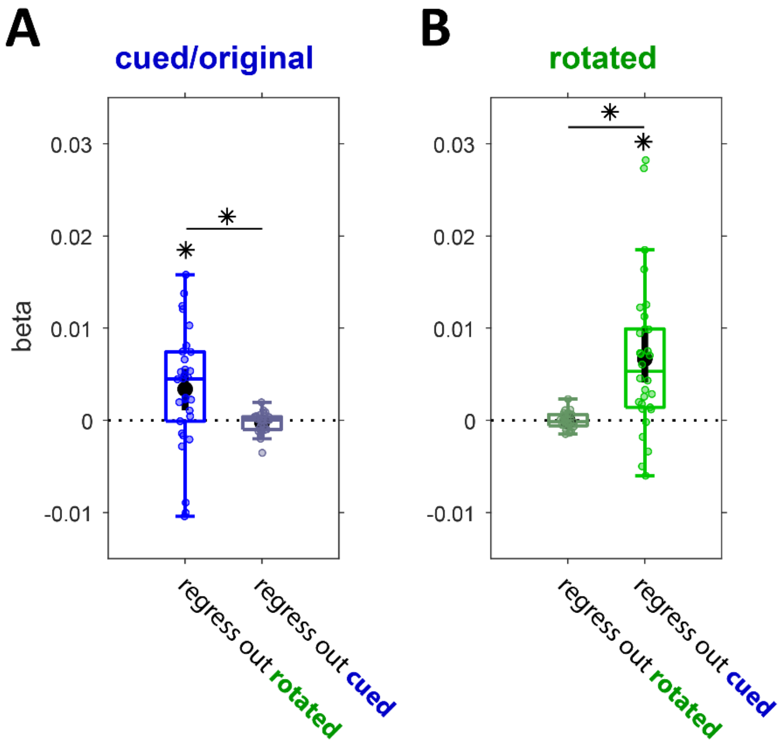

Supplemental Figure 5. Model fits of cued and rotated item models at Impulse 2 when the effect of one a specific model (cued or rotated item) is estimated from an independent half of the data, and then regressed out from the other half. **A.** Cued item model fit when independently regressing out the rotated item effect ($p < 0.01$, $BF_{10} = 12.572$), and the cued item effect ($p = 0.21$, $BF_{10} = 0.312$). Difference: $p < 0.01$, $BF_{10} = 17.094$). **B.** Rotated item model fit when independently regressing out the rotated item effect ($p = 0.7686$, $BF_{10} = 213$), and the cued item effect ($p < 0.01$, $BF_{10} = 856.518$). Difference: $p < 0.01$, $BF_{10} > 1000$).
